# Delivering a primary care review for people prescribed opioids for persistent pain: a qualitative study on the facilitators and barriers facing practice pharmacists

**DOI:** 10.3399/BJGPO.2025.0156

**Published:** 2026-04-08

**Authors:** Charlotte Woodcock, Nicola Cornwall, Sarah A Harrisson, Julie Ashworth, Lisa Dikomitis, Toby Helliwell, Eleanor Hodgson, Roger Knaggs, Christian D Mallen, Tamar Pincus, Miriam Santer, Simon White, Clare Jinks

**Affiliations:** 1 Centre for Musculoskeletal Health Research, Keele University, Keele, UK; 2 Midlands Partnership University NHS Foundation Trust, Haywood Hospital, Stoke-on-Trent, UK; 3 Warwick Medical School, University of Warwick, Coventry, UK; 4 Moorland Medical Centre, Leek, UK; 5 School of Pharmacy, University of Nottingham, Nottingham, UK; 6 Primary Integrated Community Services Ltd, Nottingham, UK; 7 Department of Psychology, Royal Holloway, University of London, Egham, UK; 8 School of Psychology, University of Southampton, Southampton, UK; 9 Primary Care Research Centre, University of Southampton, Southampton, UK; 10 School of Allied Health Professionals and Pharmacy, Keele University, Keele, UK

**Keywords:** chronic pain, general practice, opioid analgesic, pharmacist

## Abstract

**Background:**

Often, people living with persistent non-cancer pain are prescribed opioids long term, despite a lack of evidence for their long-term effectiveness and safety. This study informed the design of a new practice pharmacist-led review (the PROMPPT review) for people prescribed opioids for persistent pain in UK primary care.

**Aim:**

To explore the perspectives of pharmacists working in UK general practice regarding the proposed PROMPPT review, and to identify barriers to and facilitators of its delivery in practice, including supporting opioid deprescribing where appropriate.

**Design & setting:**

Multi-method qualitative study conducted with pharmacists working in primary care, who were recruited via professional networks predominantly in the East Midlands and West Midlands, UK.

**Method:**

Pharmacists with experience of consulting in primary care participated in semi-structured interviews (*n* = 13) and two focus groups (*n* = 16) to explore attitudes to, beliefs about, and experiences of the proposed PROMPPT review for people living with persistent pain. The Theoretical Domains Framework (TDF) provided a framework for data collection and thematic analysis. Facilitators and barriers were mapped to components of the Capability Opportunity Motivation — Behaviour (COM-B) model.

**Results:**

In total, 16 facilitators and barriers relating to the delivery of the PROMPPT review were identified across 10 domains of the TDF. Factors included access to evidence-based patient-facing resources, receiving professional colleagues’ peer support, and having a therapeutic alliance with patients. These mapped to the COM-B model components as follows: capability (knowledge, skills), opportunity (environmental context and resources, social influences), and motivation (social or professional role and identity, beliefs about capabilities, beliefs about consequences, intentions, goals, emotions).

**Conclusion:**

This study provides theoretically based evidence of factors influencing pharmacists’ delivery of the proposed PROMPPT review in relation to pharmacist capability, motivation, and opportunity. This work informed the co-design of both the intervention and the pharmacist training package.

## How this fits in

Best practice guidance recommends that patients who are prescribed long-term opioids for persistent non-cancer pain are regularly reviewed and opioids are gradually tapered if treatment goals are not met. To support implementation of this guidance, we aimed to develop a proactive review for people prescribed opioids for persistent pain — namely, the Proactive clinical Review of patients taking Opioid Medicines long term for persistent Pain led by clinical Pharmacists in primary care Teams (PROMPPT) review — to be delivered by pharmacists working in general practices. This study identified facilitators and barriers, as perceived by pharmacists, for the effective delivery of the PROMPPT review. It also highlights theoretically informed training needs, which were mapped to capability, motivation, and opportunity, to support pharmacists’ delivery of these reviews in practice.

## Introduction

Around 43% of adults in the UK are living with persistent pain,^1^ defined as lasting for ≥3 months and not caused by cancer,^2^ and are commonly treated in general practice (primary care). Opioids are often prescribed,^3,4^ despite a lack of evidence for their long-term effectiveness and the risk of adverse events.^5–8^ High initial doses, duration of prescribing after surgery, and a range of patient-level characteristics have been associated with long-term (and potentially problematic) opioid use.^9^ Guidance recommends that patients who are prescribed opioids long term for persistent pain are regularly reviewed, but limited time in routine GP appointments means this is not consistently achieved.^10–12^

Since 2016, a large expansion in the number of pharmacists working in UK general practices has seen them take on a greater role in medication reviews and the primary care management of chronic conditions.^13–18^ Systematic reviews of pharmacist-led interventions have provided some evidence of effectiveness in optimising opioid therapy and management of persistent pain, but further high quality clinical trials are needed.^19,20^ The Proactive clinical Review of patients taking Opioid Medicines long term for persistent Pain led by clinical Pharmacists in primary care Teams (PROMPPT) research programme aims to build on existing evidence and to develop a new primary care review for patients who are prescribed opioids for persistent pain to be delivered by practice pharmacists (named simply as ‘pharmacists’ hereafter) who are independent prescribers.

This study formed part of a larger qualitive programme of work informing the iterative development and co-design of the PROMPPT intervention, which comprises the review and the pharmacist training package.^21–23^ Previously published research identified anticipated facilitators and barriers of a PROMPPT review for people living with persistent pain (that is, the intervention’s intended recipients);^21^ however, understanding the perspectives of those delivering the proposed review is also important in order to maximise effectiveness.^24^ As such, to support the development of the overall intervention, this study aimed to identify barriers and facilitators perceived by pharmacists, who work clinically in primary care and are independent prescribers, in delivering the PROMPPT review. Delivery of the PROMPPT review was conceptualised as a complex behaviour that may involve:

- patients telling their pain story;
- initiating and navigating discussions about reducing opioids;
- talking about self-management;
- making referrals;
- booking follow-ups; and
- forming opioid reduction plans, where appropriate.

## Method

### Design

A multi-method qualitative study was designed, comprising pharmacist interviews and focus groups. The Theoretical Domains Framework (TDF)^25^ was used as a guiding framework for data collection and analysis. It features 14 domains, situated within the Capability Opportunity Motivation — Behaviour (COM-B) model of behaviour change,^26^ and allows for a wide range of potential influences to be considered for pharmacist delivery of the PROMPPT review.

### Recruitment

Pharmacists were eligible if they were working clinically in UK general practices and had completed independent prescriber training. Targeted recruitment was conducted through professional networks predominantly in the East Midlands and West Midlands in the UK; these were the two regions that would be the focus of a subsequent feasibility study^27^ and randomised controlled trial^28^ (Supplementary file S1). Remuneration for pharmacists’ time was offered, consistent with their professional pay band.

### Data collection

Interview and focus group topic guides drew on TDF domains and were informed by stakeholder discussions, as described elsewhere.^23^ Interviews were conducted first (between July 2019 and October 2019), and questions aimed to explore pharmacists’:

- experiences of consulting with patients;
- current approaches to opioid deprescribing; and
- views about a potential new pain review to be delivered by pharmacists in primary care (Supplementary file S2).

Interviews were conducted by NC (female) via telephone. Recruitment stopped when data saturation had been reached.^29^

Focus groups were conducted between November 2019 and January 2020 (Supplementary file S3). At this stage of the programme of work, an initial prototype of the intervention was proposed; it contained preliminary intervention components, including a patient-facing pre-consultation form and an interactive leaflet that was used as stimulus for in-depth exploration of the proposed review (Supplementary files S4 and S5).

Focus groups were facilitated by CJ (female) and CW (female). RK (male) attended the second focus group to respond to pharmacy specific queries from participants. Interviews and focus groups were digitally audio-recorded. Research team members who collected data were experienced post-doctoral qualitative researchers. NC, CJ and CW did not know participants prior to their involvement in the study. RK knew some participants in focus group 2 as professional colleagues.

### Data preparation and analysis

Audio recordings were transcribed verbatim, anonymised, checked for accuracy, and imported into NVivo (version 12) to aid data management. To ensure rigour, a phased approach was followed. Transcripts were read and re-read for familiarisation. Three members of the multidisciplinary research team (CJ, applied health expertise and trained in the person-based approach to intervention development;^30^ CW, a chartered psychologist working in applied health and behaviour change; and SW, academic pharmacy) independently coded three transcripts deductively to TDF domains.^31,32^ Regular meetings with the wider team were held to discuss initial coding decisions until consensus was reached. All remaining data was deductively coded by three researchers to TDF domains, with regular meetings to ensure a robust approach. This was followed by inductive analysis within each TDF domain to generate domain-specific subthemes.

## Results

For the interviews, 13 pharmacists were purposively sampled from 24 responses to study invitations. Although recruitment efforts focused on East Midlands and West Midlands, one participant was located in Scotland. Mean interview length = 49.60 minutes. For the focus groups, 16 pharmacists were sampled from 22 positive responses to invitations to participate and attended one of two focus groups (*n* = 7 and *n* = 9 respectively; mean length 2 hours 12 minutes).

During data collection, pharmacists drew on their experiences of primary care consultations. Some interview participants voluntarily disclosed their length of time working in general practice, which ranged from 9 months to 13 years. All interviewed participants reported conducting medication reviews, seeing people living with persistent pain, and deprescribing. In the interviews and focus groups, pharmacists talked about facilitators and barriers to delivering a proposed PROMPPT review that related to 10 of 14 TDF domains, which contained 16 subthemes; these are described below, and summarised in Table 1 and Supplementary Table S6.

**Table 1.** TDF domains, and facilitator and barrier subthemes for pharmacist delivery of a PROMPPT review

COM-B model component	TDF domain	Subtheme
Capability	Knowledge	Experience of consulting in primary care
		Knowledge about pain, pain management, and opioid deprescribing
		Patient knowledge of a PROMPPT review
	Skills	Person-centred shared decision-making
		Supporting patient pain management
Motivation	Social or professional role and identity	Part of pharmacist’s role in primary care
	Beliefs about capabilities	Confidence to deliver PROMPPT reviews
	Beliefs about consequences	Patient benefits
	Intentions	Intention to review patients
	Goals	Do what’s best for the patient
	Emotions	Pain reviews are emotionally demanding
Opportunity	Environmental context and resources	Capacity and resource to deliver PROMPPT reviews
		A consistent approach
		Lack of definitive tapering guidelines
	Social influences	Pharmacist social support
		Patient–pharmacist rapport

COM–B = Capability, Opportunity, Motivation - Behaviour. PROMPPT = Proactive clinical Review of patients taking Opioid Medicines long term for persistent Pain led by clinical Pharmacists in primary care Teams. TDF = Theoretical Domains Framework.

### Knowledge

#### Experience of consulting in primary care

Experiential knowledge gained from consulting with patients in primary care was considered to be a prerequisite for delivering a PROMPPT review. Specifically, experience of consulting with people with persistent pain was seen as desirable because these patients often had comorbidities and their cases were described as complex.

Pharmacists also needed to know the services that were available for referral, as well as the associated policies and processes.

In addition, pharmacists told us they would be uncomfortable conducting this type of review when newly qualified or if they had recently become a prescriber. What constituted ‘enough’ experience varied, although most participants agreed that 6–12 months’ experience of consulting in general practice was desirable, and that those with less experience would benefit from training.

#### Knowledge about pain, pain management, and opioid deprescribing

Being knowledgeable about the biopsychosocial nature of persistent pain was considered to be important for supporting patients.

Specialised knowledge of medicines, associated side effects, and long-term effectiveness was considered an advantage when reviewing pain medicines and optimising medication regimes.

Participants also said that having knowledge around non-pharmacological approaches was valuable in terms of being able to support patients’ pain management. Participants drew on experiential knowledge when describing their understanding of deprescribing, but some desired additional training around polypharmacy and deprescribing opioids specifically:

*‘I’d have to brush up on my clinical knowledge around what combinations and how we should go about deprescribing them so, the clinical aspects behind it, like what interventions and how do we make those interventions.’* (Focus group 1)

#### Patient knowledge of a PROMPPT review

Pharmacists suggested that it would be helpful for patients to know the role of the pharmacist and what to expect before attending a PROMPPT review. They liked the idea of a pre-consultation form that encouraged patients to consider their concerns about pain and pain medicines, suggesting it would help patients to prepare for the consultation.

### Skills

#### Person-centred shared decision-making

Most pharmacists suggested patient-centred consultation skills were needed to deliver a PROMPPT review. Skills mentioned included:

- managing patient expectations about persistent pain and pain relief;
- offering information about medicines;
- addressing concerns;
- listening to what matters most;
- understanding barriers to reducing opioids;
- showing empathy and compassion; and
- involving patients in decision-making.

As one interviewee noted:

*‘The patient needs to feel that they’ve been included* [in] *all of the decisions… they don’t want to feel like it’s happened to them, they want to feel they’ve been the one to decide things.’* (Interview, participant 19)

Participants also expressed that it is important to find out patients’ readiness to reduce opioids and to adapt the consultation accordingly:

*‘The danger we have with pharmacists is we fix things and the danger is, if you see it as a cycle of change model, we launch straight into the action phase, forgetting actually there are four steps before that.’* (Interview, participant 15)

Participants told us that the person-centred skills that are required to conduct a PROMPPT review effectively — such as facilitating patient motivation to reduce opioids — go beyond what is required for a regular medication review, and recognised a need for further training.

In contrast, some participants suggested times when a more paternalistic approach might be appropriate, for example, when patient safety was at risk or to encourage patient attendance. As an example, it was suggested that repeat prescriptions could be paused until the patient had attended a PROMPPT review.

#### Supporting patient pain management

Participants spoke about skills in developing and supporting individualised pain management and tapering plans. To do this effectively, they felt pharmacists needed to be independent prescribers, skilled in deprescribing, and able to manage patient expectations (for example, around withdrawal symptoms and the timing of doses). When plans include a tapering schedule, participants suggested any reduction be presented as a trial and the impact of making this change be regularly reviewed. As such, they emphasised the need for skills in mentoring and supporting patients to problem solve potential barriers during the process of reducing their opioids.

### Social or professional role and identity

#### Part of pharmacist’s role in primary care

Many participants saw a PROMPPT review aligning with their current professional responsibilities of medicine management and safe prescribing. As relatively new members of the primary care team, the opportunity to lead such a service was viewed favourably and something that could help to clarify their expertise to colleagues:

*‘It’s very important that pharmacists get embedded and are seen to be* [in] *the role where we will be reviewing these medications, we will be making sure that they’re prescribing safely around these areas that are such high risk.’* (Interview, participant 21)

### Beliefs about capabilities

#### Confidence to deliver PROMPPT reviews

Participants expressed variability in confidence regarding conducting PROMPPT reviews. Greater confidence was anticipated when consulting with patients who were willing to discuss their pain management and were open to opioid tapering.

Pharmacists spoke about being confident in arranging referrals and consulting with colleagues for complex cases where needed, although they were less confident in producing a plan for pain self-management.

Some pharmacists also highlighted the potential difficulty of health professionals who had initiated opioids then recommending opioid deprescribing:

*‘They think you got them there. I think I had that conversation with one of the patients and I said, “You're on this high dose of opioid”, and she said “It was a doctor from your surgery that got me here”.’* (Focus group 2)

Many pharmacists expected that not all patients would want to reduce their opioids and anticipated there being challenging conversations around tapering with patients who had been taking opioids for a long time, perceived them to be beneficial, want a pharmacological alternative, or had previous negative experiences with healthcare services:

*‘It’s difficult if they think “No, that’s my pain relief and that helps me. Leave me alone. You're not touching anything”. If they really truly believe that’s helping them, it’s quite difficult to have that conversation.’* (Focus group 2)

Participants felt their confidence could be maintained if they acknowledged that not all patients would be ready to reduce their opioids; in these instances, they felt it was important to avoid patient conflict.

### Beliefs about consequences

#### Patient benefits

Pharmacists thought a PROMPPT review could benefit patients by providing education around pain and pain management, showing patients they were valued, and identifying a named point of contact regarding pain in primary care. Further benefits anticipated were:

- deprescribing of inappropriate opioids;
- improved pain management;
- reduced adverse side effects;
- enhanced quality of life;
- improved understanding of pain and pain medicines; and
- using medicines more appropriately (for example, regarding the timing of doses).

### Intentions

#### Intention to review patients

Pharmacists suggested PROMPPT reviews should target specific patient groups because *‘there are going to be thousands of patients’* (Focus group 1), especially in larger practices situated in areas of deprivation. They told us that they would prioritise delivery of PROMPPT reviews for *‘the high-dose ones’* (Focus group 1) who were perceived to be at greatest risk, as well as those who were more likely to engage in a pain review.

### Goals

#### Do what’s best for the patient

Participants said their goal was to help improve patients’ quality of life by reviewing their current pain management and opioid therapy in relation to what was important to the individual. Some recognised that what they considered best for patients might not align with the patient perspective, so it was important to find a shared agenda to support patient engagement:

*‘Finding out what their experiences were, what they wanted from the pain, how the pain was impacting their life, what they thought of their medication, and where they wanted to go with the medication*.*’* (Interview, participant 22)

Participants said that finding a shared agenda might be challenging when patients’ expectations exceeded what a PROMPPT review could offer, such as seeking a pharmacological alternative to opioids.

### Emotions

#### Pain reviews are emotionally demanding

Pharmacists spoke of conversations with patients who were living with persistent pain leading to both positive and negative emotions, and said they could be stressful and worrisome. Some participants suggested negative emotions could be experienced long after a consultation and strategies to protect their own wellbeing were important:

*‘I mean there’s some very sad stories when you’re dealing with these people, when you listen, I mean you probably know, er, you can’t help but be affected by them.’* (Interview, participant 19)

### Environmental context and resources

#### Capacity and resource to deliver PROMPPT reviews

Time was the most discussed resource, with all interviewed participants and focus groups highlighting that PROMPPT reviews needed to be longer than the standard 10-minute consultation. Lack of time was considered a barrier to successful deprescribing and a contributing factor to prescribing. Many suggested an initial review would require 30 minutes to 1 hour, ideally delivered in person; however, they noted that follow-up appointments could be done remotely.

Follow-ups were considered to be *‘very important’* (Focus group 1) for patients to feel supported and to monitor the impact of changes to medicines, with the option for patients to contact the pharmacist between appointments if needed.

Participants also spoke about a wide range of services that might be relevant for patients, including other health professionals, smoking cessation services, a crisis team, social prescribers, wellbeing advisers, and the Citizens Advice Bureau. Potential barriers to meeting patient need were discussed, including variation in the availability of different services, wait times, and practice-level permissions to make referrals.

Participants felt that evidence-based resources that were accessible to patients (for example, available online or in print, and in different languages) would be valuable and had multiple potential benefits for building rapport, as well as supporting the delivery and receipt of information:

*‘I always find that quite useful by showing the videos and then printing out the literature, rather than just giving them a website.’* (Focus group 1)

#### Lack of definitive tapering guidelines

Pharmacists told us that, although local guidelines existed for deprescribing, there was nothing definitive. They felt a tapering template would be helpful and could reduce the risk of patients experiencing withdrawal symptoms or other negative effects.

#### A consistent approach

Participants noted that there was likely to be variation in the implementation of the PROMPPT intervention:

*‘I suppose there’s going to be variations across different practices. Some people will have really bought into it, some people want their pharmacist to do other things they perceive to be more important.’* (Interview, participant 22)

Several participants supported a consistent approach being taken within each practice to guard against mixed messaging and an unintentional reversal of plans agreed at a PROMPPT review (for example, colleagues reinstating a higher opioid dose).

They also felt that all general practice staff should be aware of the PROMPPT review and that it should be documented in the patients’ medical record. With practice-wide support, pharmacists felt they could discuss complex cases at multidisciplinary meetings or seek GP input. To facilitate a consistent approach, participants recommended embedding a clinical template into the patients’ medical record that included the review structure, key questions, and the care plan agreed.

Pharmacists also suggested that PROMPPT reviews would benefit from being an incentivised service to be prioritised by practices, and that pharmacists’ time should be protected for its consistent delivery.

### Social influences

#### Pharmacist social support

Pharmacists noted that social support for delivering PROMPPT reviews would be beneficial and help them to discuss complex cases, monitor prescriptions, support the completion of referral processes, and learn from experienced colleagues (for example, via shadowing opportunities, the sharing of best practice, and role play):

*‘*[It would be useful to have access to, or hear from] *pharmacists who have got experience dealing with these patients, telling us about different consultations, how well they’ve gone, and practising role plays and things like that, because I think that’s the most important thing.’* (Interview, participant 19)

#### Patient–pharmacist rapport

Participants shared strategies that might help them to develop and maintain patient trust and develop rapport, such as reassuring patients that consultations were confidential, introducing themselves to patients by their first name, supporting patient priorities, having honest conversations about the tapering process, and having regular follow-ups. It was noted that establishing trust and rapport with patients takes time to develop and, given the potential of the PROMPPT review to involve deprescribing, this might be challenging to achieve:

*‘The relationship, so you’re saying they’ve seen a GP for years and then they go to someone random and, if we just start talking about reduction straight away, it’s not going to go well.’* (Interview, participant 23)

## Discussion

### Summary

This study forms part of a research programme to develop a pharmacist-led review in primary care for people with persistent pain (PROMPPT review), to support opioid deprescribing where appropriate. It provides evidence regarding important factors to consider for its delivery, with 16 facilitators and barriers identified under 10 domains of the TDF. Key domains for successful delivery related to the COM-B model’s components of capability (knowledge, skills), motivation (social or professional role and identity, beliefs about capabilities, beliefs about consequences, intentions goals, emotions), and opportunity (environmental context and resources, social influences). Overall, pharmacists perceived multiple benefits for delivering a PROMPPT review, which aligned well with current knowledge and skills; however gaps in expertise specific to having opioid deprescribing conversations were identified, as was a need for further resources and social support.

### Strengths and limitations

To the best of our knowledge, this is the first study that has focused on practice pharmacists’ views on delivering a review to support opioid deprescribing where appropriate in UK primary care. This study has identified factors likely to influence how a review is delivered from the perspective of potential deliverers, a feature of intervention development that is not often reported. This is important when intervention effectiveness is dependent on the effective use of the relevant knowledge and skills of those delivering it.

Another key strength was the use of multiple methods. Interviews allowed pharmacists to consider and share their views and clinical experiences in depth. Focus groups enabled pharmacists’ experiences to be heard, clarified, and expanded on; as an example, focus group participants would often ask follow-up questions to another participant’s answer. Data analysis followed a robust systematic approach using an established theoretical framework, and multidisciplinary team discussions ensured critical consideration of key factors.

Our study does have some limitations, however, that should be acknowledged. Participants predominantly worked in only two regions of the UK (East Midlands and West Midlands) and responded to recruitment advertisements; as such, they may not be representative of all practice pharmacists working in primary care. We did not systematically collect participant demographic characteristics, although some participants reported their length of experience in general practice and these data suggest a broad range was represented. Following data collection, participants did not have an opportunity to review transcripts or findings for comment or correction. However, multidisciplinary stakeholder meetings, which included pharmacists, critically discussed the main findings as part of the intervention development process, details of which have been published elsewhere.^23^

### Comparison with existing literature

The present study supports previous research that considered the patients’ perspectives of a PROMPPT review.^21^ It also goes beyond consideration of pharmacist knowledge and skills; these are two domains that have dominated previous practitioner deprescribing interventions and have been deemed insufficient when supporting practitioners, such as pharmacists, to change their behaviour when consulting with patients.^33^

A consistent finding between patients and pharmacists is the importance of building a productive patient–pharmacist relationship that relies on communication skills to support a person-centred approach to shared decision-making.^21^ Specifically, pharmacists’ skills at expressing empathy, letting a patient know they are believed and cared for, as well as actively listening to patients’ concerns, have all been identified as contributing towards the development of rapport and mutual trust, which is considered key for engaging patients in conversations about reducing their opioids.^34^ Research suggests that practitioners have concerns that deprescribing conversations can harm rapport with patients as these are often difficult or challenging.^11,35–38^ The pharmacist working in a primary care setting is an emerging role and some may not yet have a strong therapeutic alliance with their patients;^35^ this study reinforces the importance of nurturing these alliances from a pharmacist perspective, and indicates that pharmacists may benefit from training on communication skills to support person-centred conversations for discussing an opioid reduction.

Research evidence has highlighted the challenges of having opioid deprescribing discussions and our study has shown that pharmacists share these concerns, especially those relating to a lack of tapering guidelines^31^ and variation in the availability of resources.^11^ Pharmacists in our study also highlighted the potential emotional burden of these conversations, a negative impact that has previously been cited by GPs^11^ and advanced nurse practitioners.^39^

Perceived resistance from patients is another reason conversations around opioid deprescribing may be considered challenging. Our study supports the view that patients are expected to vary in their levels of engagement, which has been a consistent finding across multiple studies: patient resistance has been linked to fear of pain, withdrawal, or a belief that opioids are necessary to maintain quality of life.^21,40,41^ Patient motivation to engage is key and often reliant on personal goals. A recent systematic review recommended that tapering plans consider the context of a patient’s life;^36^ our study also suggests that individualised pain management support should align with what matters to patients. It has been found that individualised pain management support is likely to involve identifying a patient’s current stage of change (pre-contemplation, contemplation, preparation, action, or maintenance) and adapting the consultation accordingly;^42^ however, our findings highlight pharmacists’ tendency to consult in the action phase, which may explain why practitioners expressed being more confident about consulting with patients who are ready to make an opioid reduction. Although pharmacists recognised the importance of working with pre-contemplative and contemplative patients to support them in progressing towards action, they also acknowledged that further training might be needed to work confidently in those earlier stages.

### Implications for research and practice

The study has identified some important associated training needs that might support pharmacists’ capability, opportunity, and motivation to deliver PROMPPT reviews. Findings point to supporting pharmacists’ confidence and ability to cope with the emotional burden, and developing a therapeutic alliance for shared decision-making. Strategies need to be developed to help pharmacists cope with patient disclosure in order to protect their own wellbeing. Strategies need to be developed to help pharmacists cope and protect their own wellbeing in instances where patients disclose potentially distressing information.

It has been highlighted elsewhere that the pharmacist role in the primary care setting is relatively new and evolving,^18^ and a pharmacist-led review could help to develop their position within the primary care workforce.^43,44^ This study suggests that delivery of a PROMPPT review aligns well with pharmacist expertise. Since this work was completed, pharmacists have played a significant role in delivering structured medication reviews (SMRs) for patients who have been prescribed dependence-forming medicines.^45^ Guidance suggests SMRs provide individualised care involving shared decision-making.^46^ This study suggests additional training and resources are required to support pharmacist capability and motivation in supporting patient-centred conversations, especially in cases where there is perceived to be a degree of patient resistance around deprescribing.

The facilitators and barriers to delivering a PROMPPT review, as perceived by pharmacists in our study, correspond to key delivery features previously identified by patients, and informed the co-design of the intervention and associated training package ahead of a feasibility study.^21,26,28^ A cluster randomised controlled trial is currently underway to evaluate the clinical and cost effectiveness of the PROMPPT intervention.^27^

## Trial registration

researchregistry4796

## Data

The data supporting the findings of this study are available from the corresponding author on reasonable request and if in line with participant consent for data use.
